# Potato starch quality in relation to the treatments and long-term storage of tubers

**DOI:** 10.1038/s41598-025-85657-0

**Published:** 2025-02-03

**Authors:** Katarzyna Brążkiewicz, Jarosław Pobereżny, Elżbieta Wszelaczyńska, Bożena Bogucka

**Affiliations:** 1 Institute of Agri-Foodstuff Commodity, Bydgoszcz University of Science and Technology, 7 Kaliskiego St, 85-796 Bydgoszcz, Poland; 2 Department of Agrotechnology and Agribusiness, University of Warmia and Mazury in Olsztyn, 8 Oczapowskiego St, 10-719 Olsztyn, Poland

**Keywords:** Environmental impact, Agroecology

## Abstract

Starch is the most important component of potato tubers, the structure and composition of which play a key role in their utilization as well as processing and storage. Potato seed treatment may play the biggest role in ensuring proper plant growth and development. Isolated starch from tubers of different potato varieties for food processing was evaluated immediately after harvesting and after long-term storage with consideration of different products for potato seed treatment. The quality of starch was evaluated in terms of starch grain size, total starch content, pH, starch stability after freezing, gelatinization temperature as well as phosphorus and amylose content. The varieties differed significantly in starch content and quality. The Beo variety had the highest starch content in dry tuber weight (77.8%) and the best starch quality characteristics (the lowest starch stability after freezing − 18.0% and highest gelatinization temperature − 64.0 ℃ onset and 68.4 ℃ end). Simultaneous treatment of tubers with Supporter and Moncut 460 SC contributed to the highest starch content in tubers (77.6% d. m.) as well as increasing its stability after freezing (20.8%) and decreasing its gelatinization temperatures (61.7 ℃ onset and 66.0 ℃ end). This may be due to the increased proportion of large starch grains and higher amylose and phosphorus content. A slight decrease in starch quality traits was shown after long-term storage of tubers. Maintaining constant conditions during storage along with the applied treatments contributed to this. It is recommended to use products for potato seed treatment in the production technology of potato for consumption and for starch production.

## Introduction

Thanks to its high nutritional content (carbohydrates, protein, minerals), the potato (*Solanum tuberosum* L.) continues to rank among the most important crops for feeding the world’s population^1–2^. It also owes its strong market position to its wide range of uses. Potatoes are mainly destined for direct consumption and for food processing into French fries, chips, dries and alcohols or feeds^3^. Potato starch production is dominant, as it is the third most important raw material used in the food and non-food sectors of global production^4^.

Starch is the main carbohydrate found in the potato, and the most important component of the tuber’s dry weight^5–6^. It is composed of two polysaccharide components: amylose (15–30%) and amylopectin (70–85%), the ratio of which depends on genetic determinants^7–8^. Both of these components have a chain structure. Amylose units are linked by α-1,4- glycosidic bonds and have a linear arrangement, while amylopectin units are branched via α-1,6-glycosidic bonds to form side branches occurring every 22–77 glucose units^6,9^. Moreover, the structure of amylose is long and narrow, and its degree of polymerization depends on its source^8,10^. Due to its low production costs, easy availability and unique physicochemical and morphological properties as compared to other commercially available starches, potato starch is used in the food industry on a large scale^11–13^. Its favorable characteristics are, among other things, due to its large granule size, purity, neutral taste, relatively long amylose and amylopectin chains, the presence of phosphate ester groups on amylopectin, and its ability to form transparent gels when heated^11,14,15^. The natural properties of potato starch are used in the food industry to give products the desired texture, appearance, consistency, density and stability during long-term storage^13,15,16^. Due to the existence of three active hydroxyl groups on glucose molecules, chemical modifications can be carried out to increase its hydrophobicity^8^.

The overall quality of starch is a difficult issue to define considering the wide range of its characteristics - pH, grain size, amylose and phosphorus content, gelatinization temperature and starch stability. Its content in potato tubers and quality depend on many factors: genetic and environmental conditions (place of cultivation, temperature and amount of precipitation, date of harvest), as well as applied agrotechnology^10,11,17–19^. In the technology of starch potato cultivation, mineral fertilization, the type of pesticides used and preparations for plant growth and development, including growth modulators, are of great importance. The use of such agents is aimed at supporting the development of the root system, especially in the initial vegetation period, as well as at increasing resistance to abiotic environmental factors and stress. In addition, it accelerates the intensity of plant growth and development by modulating the chlorophyll content having a significant impact on the process of photosynthesis, which plays a special role in the synthesis of starch in potato tubers^8,18,20–25^. The process of tuber storage also has a direct impact on the content and quality of potato starch^9,20^. During storage, changes in starch quality can occur as a result of respiration, transpiration and germination processes^9,26^. During tuber storage the biochemical process of potato starch decomposition occurs and the intensity of these changes depends on temperature and storage time. In a stored potato tuber, hydrolysis of the starch chain takes place, resulting in a mixture of dextrin, maltose and glucose^27^. At low temperatures, there is also a breakdown of starch to sucrose, which is eventually metabolically broken down by invertase to glucose and fructose^27,28^. This is particularly important for potato varieties intended for processing into chips and French fries, as it is the monosaccharide content that is most responsible for the quality of fried products^9,27,28^.

With the continuous development of potato cultivation and storage technologies involving the introduction of new formulations, knowledge of the physical, chemical and functional properties of starch can ensure the consistent and desirable quality of starch for the food industry, and will also provide information for growers in developing proper post-harvest potato management^3,4,10–13,17−19^.

It should also be remembered that unfavorable physicochemical changes in starch also occur during processing that use high temperatures (above 150 ℃)^17^.

The purpose of this study was to determine the effects of treatments and long-term storage on the starch quality of potato varieties for food processing.

## Results and discussion

### Starch content in the dry matter of the tubers

This study showed that the percentage of starch in the dry weight of tubers significantly depended on all the factors used, both in the first and second years of the study. The varieties tested differed significantly in starch content in tuber dry matter, which ranged from 74.0–77.6% immediately after harvest and from 73.6 to 77.2% after storage (Tables 1 and 2). The results obtained are in line with the report of Lu et al.^29^, who note that the starch content in the dry matter of tubers ranges from 67.0 to 79.0%. The decrease in starch content after storage shown in this study may be due to the physiological processes taking place - transpiration and respiration. The extent of these decreases depends partly on the storage temperature and, more importantly, on the variety. Tubers of different varieties have different thickness of periderm that determines the intensity of transpiration processes, and on the amount of deposited suberin, which is a natural barrier to water transport^5,20,30,31^. The ‘Beo’ variety showed the highest starch content both after harvest and after storage, followed by ‘Picus’ and ‘Pirol’ (Tables 1 and 2). The differences in starch content are due to genetic conditions and the agrotechnology used at the time of cultivation and to the variability of the dry matter of the tested varieties intended for processing, since starch content depends in direct proportion on the dry matter content of the potato tubers^19,32,33^. In addition, for potatoes destined for processing, especially for refined products, the starch content which should be above 20% in fresh weight, i.e. 75% in dry weight of tubers, is important^28,34^. Our results are consistent with the studies of the above authors (Tables 1 and 2). It was noted that higher starch content in dry matter (Tables 1 and 2) was characteristic of tubers from the first year of the experiment − 2021, which is a consequence of more favorable weather conditions^35,36^ - higher average temperature and total precipitation during the potato growing season (Tables 3 and 4 ). This is consistent with reports by Dahal et al.^37^, who indicate that the optimal temperature for photosynthesis and biomass accumulation in potatoes is around 20 ℃. Rymuza et al.^38^, studying nine potato varieties, showed that the starch content in tubers depends on precipitation during the growing season. The authors also reported on the diversified response of varieties to weather conditions in different growing seasons in terms of starch accumulation in tubers.

**Table 1:** Starch content in the dry weight of potato tubers, starch grain size and total starch content in the tested potato varieties depending on the treatment and evaluation date in 2021.

Variety	Treatment	Starch content in dry weight of potato tubers [%]		Starch grain size (weighted average) [%]		Total starch content [%]	
		AH*	AS**	AH	AS	AH	AS
BEO	Control	77.0 ± 0.2	76.7 ± 0.2	27.16 ± 0.02	26.88 ± 0.03	86.3 ± 0.3	83.4 ± 0.4
	Supporter	77.4 ± 0.2	76.8 ± 0.2	27.29 ± 0.04	27.00 ± 0.03	87.2 ± 0.3	84.1 ± 0.4
	Moncut 460 SC	77.8 ± 0.3	77.3 ± 0.3	27.36 ± 0.05	27.04 ± 0.04	91.7 ± 0.3	86.4 ± 0.2
	Supporter +Moncut 460 SC	79.0 ± 0.2	78.7 ± 0.2	27.54 ± 0.07	27.14 ± 0.03	95.9 ± 0.4	87.9 ± 0.6
**Average**		**77.8 ± 0.8**	**77.4 ± 0.8**	**27.33 ± 0.15**	**27.02 ± 0.10**	**90.3 ± 4.0**	**85.5 ± 1.9**
PIROL	Control	72.0 ± 0.2	71.5 ± 0.2	24.20 ± 0.07	23.92 ± 0.02	83.6 ± 0.3	80.5 ± 0.4
	Supporter	72.5 ± 0.2	72.1 ± 0.2	24.56 ± 0.03	24.02 ± 0.02	84.5 ± 0.2	82.2 ± 0.1
	Moncut 460 SC	75.6 ± 0.2	75.3 ± 0.2	24.60 ± 0.03	24.14 ± 0.04	85.8 ± 0.6	82.6 ± 0.4
	Supporter +Moncut 460 SC	75.9 ± 0.2	75.2 ± 0.2	24.70 ± 0.07	24.32 ± 0.07	86.4 ± 0.3	82.1 ± 0.3
**Average**		**74.0 ± 1.8**	**73.5 ± 1.8**	**24.52 ± 0.20**	**24.10 ± 0.16**	**85.1 ± 1.2**	**81.9 ± 0.9**
PICUS	Control	74.7 ± 0,1	74.3 ± 0.2	25.78 ± 0.08	25.50 ± 0.06	86.8 ± 0.4	82.2 ± 0.3
	Supporter	75.6 ± 0.2	75.3 ± 0.1	26.00 ± 0.05	25.54 ± 0.04	87.8 ± 0.3	82.7 ± 0.4
	Moncut 460 SC	76.0 ± 0.1	75.8 ± 0.2	26.04 ± 0.07	25.88 ± 0.06	90.1 ± 0.3	83.6 ± 0.3
	Supporter +Moncut 460 SC	77.8 ± 0.2	77.5 ± 0.1	26.18 ± 0.07	26.02 ± 0.11	91.1 ± 0.4	88.3 ± 0.3
**Average**		**76.0 ± 1.2**	**75.7 ± 1.2**	**26.00 ± 0.16**	**25.74 ± 0.24**	**89.0 ± 1.8**	**84.2 ± 2.5**
**Averages for treatments**	Control	74.6 ± 2.2	74.2 ± 2.3	25.71 ± 1.29	25.43 ± 1.29	85.6 ± 1.5	82.0 ± 1.3
	Supporter	75.2 ± 2.1	74.7 ± 2.1	25.95 ± 1.18	25.52 ± 1.29	86.5 ± 1.5	83.0 ± 0.9
	Moncut 460 SC	76.5 ± 1.0	76.1 ± 0.9	26.00 ± 1.19	25.69 ± 1.26	89.2 ± 2.7	84.2 ± 1.7
	Supporter + Moncut 460 SC	77.6 ± 1.4	77.1 ± 1.5	26.14 ± 1.23	25.83 ± 1.23	91.1 ± 4.2	86.1 ± 3.0
**Average for the experiment**		**75.9 ± 2.0**	**75.5 ± 2.1**	**25.95 ± 1.21**	**25.62 ± 1.23**	**88.1 ± 3.4**	**83.8 ± 2.4**
^1^LSD α = 0.05		^2^A = 0.106; B = 0.088; C = 0.089; B/A = 0.124; A/B = 0.144; C/A = ^3^N.S.; A/C = N.S.; C/B = 0.155; B/C = 0.160		A = 0.029; B = 0.038; C = 0.052; B/A = 0.054; A/B = 0.052; C/A = 0.073; A/C = 0.069; C/B = 0.090; B/C = 0.087		A = 0.102; B = 0.318; C = 0.311; B/A = 0.449; A/B = 0.379; C/A = 0.440; A/C = 0.393; C/B = 0.539; B/C = 0.564	

^*^AH - Directly after harvest, **AS - After 6 months of storage.

^1^LSD - least significant difference, ^2^Experimental factors: A – Evaluation date, B – Variety, C –Treatment application, ^3^not significant.

**Table 2:** Starch content in the dry weight of potato tubers, starch grain size and total starch content in the tested potato varieties depending on the treatment and evaluation date in 2022.

Variety	Treatment	Starch content in dry weight of potato tubers [%]		Starch grain size (weighted average) [%]		Total starch content [%]	
		AH*	AS**	AH	AS	AH	AS
BEO	Control	76.4 ± 0.2	76.0 ± 0.2	26.88 ± 0.02	26.60 ± 0.04	86.5 ± 0.3	83.5 ± 0.6
	Supporter	76.9 ± 0.2	76.4 ± 0.2	27.03 ± 0.07	26.75 ± 0.04	86.6 ± 0.4	83.4 ± 0.4
	Moncut 460 SC	77.3 ± 0.1	76.9 ± 0.2	27.10 ± 0.05	26.78 ± 0.06	91.9 ± 0.4	86.5 ± 0.3
	Supporter +Moncut 460 SC	78.5 ± 0.2	78.2 ± 0.2	27.26 ± 0.06	26.90 ± 0.04	95.3 ± 0.3	87.2 ± 0.3
**Average**		**77.3 ± 0.8**	**76.9 ± 0.9**	**27.10 ± 0.15**	**26.80 ± 0.12**	**90.1 ± 3.9**	**85.2 ± 1.8**
PIROL	Control	72.5 ± 0.2	72.0 ± 0.2	23.94 ± 0.05	23.78 ± 0.04	83.8 ± 0.4	80.6 ± 0.5
	Supporter	72.7 ± 0.2	72.5 ± 0.1	24.30 ± 0.06	23.88 ± 0.05	83.9 ± 0.3	81.5 ± 0.3
	Moncut 460 SC	74.8 ± 0.2	74.6 ± 0.2	24.34 ± 0.05	23.90 ± 0.07	86.0 ± 0.3	82.7 ± 0.3
	Supporter +Moncut 460 SC	76.1 ± 0.2	75.5 ± 0.2	24.44 ± 0.07	24.18 ± 0.11	85.8 ± 0.2	82.2 ± 0.3
**Average**		**74.0 ± 1.6**	**73.6 ± 1.5**	**24.30 ± 0.21**	**23.90 ± 0.17**	**84.9 ± 1.1**	**81.8 ± 0.9**
PICUS	Control	74.9 ± 0,2	74.0 ± 0.3	25.52 ± 0.06	25.25 ± 0.04	87.0 ± 0.3	82.3 ± 0.2
	Supporter	75.1 ± 0,2	74.1 ± 0.2	25.74 ± 0.05	25.30 ± 0.06	87.2 ± 0.4	82.8 ± 0.4
	Moncut 460 SC	75.6 ± 0.1	74.5 ± 0.2	25.80 ± 0.05	25.60 ± 0.04	90.3 ± 0.2	83.7 ± 0.2
	Supporter +Moncut 460 SC	77.7 ± 0.3	76.7 ± 0.2	25.94 ± 0.11	25.78 ± 0.02	90.3 ± 0.4	87.4 ± 0.2
**Average**		**75.8 ± 1.2**	**74.8 ± 1.2**	**25.80 ± 0.17**	**25.50 ± 0.23**	**88.7 ± 1.7**	**84.1 ± 2.1**
**Averages for treatments**	Control	74.6 ± 1.7	74.0 ± 1.7	25.40 ± 1.28	25.20 ± 1.22	85.8 ± 1.5	82.1 ± 1.3
	Supporter	74.9 ± 1.8	74.3 ± 1.7	25.70 ± 1.18	25.30 ± 1.24	85.9 ± 1.6	82.6 ± 0.9
	Moncut 460 SC	75.9 ± 1.1	75.3 ± 1.2	25.70 ± 1.20	25.40 ± 1.25	89.4 ± 2.7	84.3 ± 1.7
	Supporter +Moncut 460 SC	77.4 ± 1.1	76.8 ± ± 1.2	25.90 ± 1.22	25.60 ± 1.19	90.5 ± 4.1	85.6 ± 2.6
**Average for the experiment**		**75.7 ± 1.8**	**75.1 ± 1.8**	**25.70 ± 1.24**	**25.40 ± 1.25**	**87.9 ± 3.3**	**83.7 ± 2.2**
^1^LSD α = 0.05		^2^A = 0.177; B = 0.110; C = 0.092; B/A = 0.156; A/B = 0.214; C/A = ^3^N.S.; A/C = N.S.; C/B = 0.159; B/C = 0.177		A = 0.046; B = 0.048; C = 0.052; B/A = N.S.; A/B = N.S.; C/A = 0.074; A/C = 0.077; C/B = 0.090; B/C = 0.092		A = 0.259; B = 0.197; C = 0.303; B/A = 0.279; A/B = 0.338; C/A = 0.428; A/C = 0.443; C/B = 0.524; B/C = 0.495	

^*^AH - Directly after harvest, **AS - After 6 months of storage.

^1^LSD - least significant difference, ^2^Experimental factors: A – Evaluation date, B – Variety, C –Treatment application, ^3^not significant.

**Table 3:** Average air temperature during potato cultivation (2021, 2022).

Year	2021				2022			
Month	Decade			Monthly average	Decade			Monthly average
	I	II	III		I	II	III	
	Average daily air temperatures (°C)				Average daily air temperatures (°C)			
May	7.5	14.0	11.3	10.9	10.3	12.6	15.8	11.2
June	16.8	19.0	20.4	18.7	15.6	15.8	19.9	17.1
July	21.0	21.4	20.1	20.8	17.6	16.4	18.2	17.4
August	17.2	17.5	14.7	16.4	18.1	22.1	19.8	20.0
September	13.2	13.4	10.6	12.4	11.4	11.4	9.5	10.8
	Average			15.8	Average			15.3

**Table 4:** Total precipitation during potato cultivation (2021, 2022).

Year	2021				2022			
Month	Decade			Total per month	Decade			Total per month
	I	II	III		I	II	III	
	Total rainfall (mm)				Total rainfall (mm)			
May	60.0	9.5	15.5	85.0	3.3	11.6	33.2	48.1
June	2.8	11.6	55.4	69.8	33.4	40.3	30.7	104.4
July	94.6	70.6	13.2	178.4	25.8	17.8	19.7	63.3
August	75.7	10.9	49.2	136.4	0.2	7.6	21.8	29.6
September	1.3	8.9	14.8	25.0	6.9	7.8	37.8	52.5
	Total during the growing season			494.6	Total during the growing season			297.7

Furthermore, the Supporter and Moncut 460 SC treatments used in this study significantly changed the starch content in tubers of the tested varieties (Tables 1 and 2). Each of the applied products, regardless of the year of the study, increased the starch content by an average of 0.5% points (pp) - Supporter, and 1.6 pp - Moncut 460 SC. However, the most favorable effect in this regard was observed after their combined application, as an increase in the starch content of tuber dry matter by an average of 2.9 pp was obtained regardless of the year and date of evaluation. The positive effect of potato growth promoters on the starch content in their tubers is also reported by^25,36,39^. According to these authors, the positive effect of growth stimulators is related to their impact on reducing the leaching of nutrients from the soil, as well as increasing the efficiency of uptake and utilization of macronutrients such as nitrogen, phosphorus and potassium.

There is a general perception that the amount of starch contained in potato tubers, especially in varieties intended for processing, affects its quality characteristics^16.35,40^. Šimková et al.^35^ report that higher starch content contributes to faster gelatinization, which is confirmed by the correlation coefficients between starch content and onset gelatinization temperature obtained in our study: *r*=-0.655 after harvest and *r*=-0.690 after storage (Tables 5 and 6). According to many authors^16,35,41^ the starch in potatoes intended for processing should have a greater proportion of grains between 20 and 60 μm in size. Moreover, potatoes with higher starch content are characterized by a higher proportion of starch grains of larger sizes. This correlation was confirmed in this study, as the correlation coefficient between starch content and its grain size was *r* = 0.624 after harvest and *r* = 0.622 after storage (Tables 5 and 6). This may, in consequence, directly affect the quality of fried products.

**Table 5:** The correlation coefficients (r) according to the rank order of Spearman between the studied parameters after harvest.

	Starch in d.m.	pH	Total starch content	Starch grain size	Amylose content	Phosphorus content	Onset gelatinization temperature	End gelatinization temperature
pH	0.256*							
Total starch content	0.609**	0.427**						
Starch grain size	0.624	0.424	0.515					
Amylose content	N.S.#	N.S.	0.227	N.S.				
Phosphorus content	0.598	N.S.	0.581	0.777	N.S.			
Onset gelatinization temperature	-0.655	-0.230	-0.678	-0.536	-0.244	-0.538		
End gelatinization temperature	-0.528	-0.260	-0.865	-0.433	-0.251	-0.487	0.654	
Starch stability after thawing	-0.737	-0.244	-0.589	-0.686	-0.138	-0.659	0.659	0.480

* indicates that the correlation is significant at the 0.05 probability level. >0.205.

** indicates that the correlation is significant at the 0.01 probability level. >0.267.

# not significant

**Table 6:** The correlation coefficients (r) according to the rank order of Spearman between the studied parameters after long term storage.

	Starch in d.m.	pH	Total starch content	Starch grain size	Amylose content	Phosphorus content	Onset gelatinization temperature	End gelatinization temperature
pH	0.692**							
Total starch content	0.646	0.593						
Starch grain size	0.622	0.641	0.518					
Amylose content	N.S.#	N.S.	N.S.	N.S.				
Phosphorus content	0.628	0.681	0.549	0.785	N.S.			
Onset gelatinization temperature	-0.690	-0.670	-0.545	-0.585	N.S.	-0.862		
End gelatinization temperature	-0.624	-0.586	-0.625	-0.395	N.S.	-0.431	0.622	
Starch stability after thawing	-0.712	-0.633	-0.651	-0.642	N.S.	-0.621	0.576	0.547

* indicates that the correlation is significant at the 0.05 probability level. >0.205.

** indicates that the correlation is significant at the 0.01 probability level. >0.267.

# not significant

## Starch grain size

In this study, the size of starch grains significantly depended on the genotype both after harvest and after storage (Tables 1 and 2). The proportion of large starch grains obtained in the first year of the study was greater overall compared to the second year. This may be due to more favorable weather conditions. In both the first and second years of the study, the largest proportion of large grains after harvest characterized the starch of the Beo variety (weighted average: 27.33, 27.10%, respectively), while the smallest was that of the Pirol variety (weighted average: 24.52, 24.30%, respectively) (Tables 1 and 2). The influence of genetic determinants on starch grain size has also been reported^41–43^. Romano et al.^42^ studying 21 commercial potato varieties (for processing and consumption) grown in Italy showed significant differences in starch grain size. In their study, differences in starch grain size were obtained depending on the genetic conditions of the potato and its intended use. In the case of potato varieties intended for processing grain size is of particular importance, since starch grains with a large diameter absorb more water which can result in increased moisture content in fried products^9^. Regardless of the starch evaluation date, each of the applied products for potato seed treatment had a significant effect on the size of starch grains (Tables 1 and 2). The fraction of the largest starch grains increased after application of the treatments, an effect that was most noticeable after the combined application of the products. Modulators, by increasing the starch content in tubers, have a direct effect on increasing the proportion of large starch grains. This is confirmed by the correlation coefficients obtained between starch content and starch grain size in this study, as described above (Tables 5 and 6). It should be noted that information on the effect of modulators on potato starch grain size is very limited. Our previous research confirmed the effect of biostimulant application on the growth of the large grain fraction^20^. However, that study concerned the use of a soil fertilizer, not products for potato seed treatment.

In each year, studied potato varieties responded with a decrease in the amount of the largest grains after long-term storage of the tubers by an average of 0.32 pp (Tables 1 and 2). The influence of storage on this starch quality parameter has been reported by other authors^5,9,44^. In the studies conducted by the mentioned authors, the magnitude of these changes depended on the storage time and storage temperature of the tubers. When tubers are stored at higher temperatures, starch is more rapidly converted to sugar and used for respiration due to changes in enzyme activity^5,45,46^. On the other hand, Pobereżny et al.^9^, while storing potatoes for the production of French fries, obtained a reduction in the fraction of large grains after each tuber storage term (3, 6 and 9 months).

## Total starch content and acidity (pH)

The results obtained in the study indicate that total starch content and starch acidity depended significantly on all experimental factors (Tables 1, 2, 7 and 8). In each year of the study, the starch obtained from tubers of the Beo variety had the highest total starch content and pH value, averaging 90.2% and 8.3, respectively. Pobereżny et al.^17^ state that potato varieties for processing differ considerably in total starch content. In addition, the authors showed that the total starch content for the Pirol variety was much higher than in this study, indicating that this starch trait is influenced by the potato cultivation technology used. In the current study, the application of potato growth-stimulating treatments resulted in a significant increase in total starch and a favorable reduction in starch acidity (an increase in pH) (Tables 1, 2, 7 and 8). Starch parameters that are directly related to acidity are amylose and phosphorus content^47^. In this study, the calculated correlation coefficients do not confirm such dependencies (Tables 5 and 6). This difference may be due to the botanical source of the starches studied as Tang and Liu^47^ determined the quality parameters of corn starch. The difference in the values of starch quality parameters depending on its origin is also reported by Trithavisup and Charoenrein^48^ as well as dos Santos et al.^49^. A parameter that significantly affects the acidity of starch is its grain size^9^. This is confirmed by the highly significant correlation coefficients obtained in this study both immediately after harvest and after long-term storage (*r* = 0.424 and *r* = 0.641, respectively) (Tables 5 and 6). The higher correlation coefficient obtained after storage may be due to the degradation process of starch grains during long-term storage^5,9,45,46^.

**Table 7:** Acidity of starch and its stability after thawing depending on treatments and evaluation date in 2021.

Variety	Treatment	pH [contractual units]		Starch stability after thawing [%]	
		AH*	AS**	AH	AS
BEO	Control	8.20 ± 0.18	7.73 ± 0.05	21.5 ± 0.3	28.4 ± 0.3
	Supporter	8.30 ± 0.20	7.72 ± 0.07	19.4 ± 0.3	28.5 ± 0.2
	Moncut 460 SC	8.30 ± 0.20	7.81 ± 0.03	15.7 ± 0.3	22.7 ± 0.2
	Supporter + Moncut 460 SC	8.79 ± 0.19	7.92 ± 0.03	15.4 ± 0.2	20.0 ± 0.5
**Average**		**7.80 ± 0.10**	**8.40 ± 0.30**	**18.0 ± 2.7**	**24.9 ± 3.8**
PIROL	Control	8.00 ± 0.22	7.43 ± 0.03	36.9 ± 0.4	41.5 ± 0.9
	Supporter	8.36 ± 0.20	7.56 ± 0.01	34.5 ± 0.3	37.1 ± 0.3
	Moncut 460 SC	8.00 ± 0.22	7.62 ± 0.01	31.6 ± 0.2	35.2 ± 0.4
	Supporter +Moncut 460 SC	8.43 ± 0.17	7.79 ± 0.01	23.8 ± 0.3	33.5 ± 0.2
**Average**		**7.60 ± 0.12**	**8.20 ± 0.28**	**31.7 ± 5.2**	**36.8 ± 3.1**
PICUS	Control	7.70 ± 0.19	7.41 ± 0.01	29.5 ± 0.1	37.7 ± 0.5
	Supporter	8.32 ± 0.19	7.51 ± 0.05	28.2 ± 0.3	35.5 ± 0.5
	Moncut 460 SC	8.05 ± 0.18	7.49 ± 0.09	25.2 ± 0.2	34.3 ± 1.0
	Supporter +Moncut 460 SC	8.62 ± 0.21	7.60 ± 0.12	23.3 ± 0.2	28.9 ± 0.5
**Average**		**7.50 ± 0.09**	**8.20 ± 0.39**	**26.6 ± 2.6**	**34.1 ± 3.4**
**Averages for treatments**	Control	7.97 ± 0.22	7.52 ± 0.17	29.3 ± 6.7	35.9 ± 5.9
	Supporter	8.33 ± 0.19	7.60 ± 0.13	27.4 ± 6.6	33.7 ± 4.0
	Moncut 460 SC	8.12 ± 0.22	7.64 ± 0.13	24.2 ± 6.9	30.7 ± 6.1
	Supporter +Moncut 460 SC	8.61 ± 0.24	7.77 ± 0.12	20.8 ± 4.1	27.5 ± 6.0
**Average for the experiment**		**7.63 ± 0.16**	**8.26 ± 0.33**	**25.4 ± 6.8**	**31.9 ± 6.2**
^1^LSD α = 0.05		^2^A = 0.475; B = 0.026; C = 0.042; B/A = 0.037; A/B = 0.476; C/A = 0.059; A/C = 0.477; C/B = 0.072; B/C = 0.068		A = 0.353; B = 0.384; C = 0.419; B/A = 0.543; A/B = 0.557; C/A = ^3^N.S.; A/C = N.S.; C/B = 0.726; B/C = 0.737	

^*^AH - Directly after harvest, **AS - After 6 months of storage.

^1^LSD - least significant difference, ^2^Experimental factors: A – Evaluation date, B – Variety, C –Treatment application, ^3^not significant.

**Table 8:** Acidity of starch and its stability after thawing depending on treatments and evaluation date in 2022.

Variety	Treatment	pH [contractual units]		Starch stability after thawing[%]	
		AH*	AS**	AH	AS
BEO	Control	8.22 ± 0.22	7.68 ± 0.03	22.1 ± 0.3	28.8 ± 0.3
	Supporter	8.30 ± 0.20	7.69 ± 0.04	20.0 ± 0.3	28.1 ± 0.2
	Moncut 460 SC	8.36 ± 0.20	7.77 ± 0.04	16.3 ± 0.3	23.0 ± 0.2
	Supporter +Moncut 460 SC	7.90 ± 0.23	7.80 ± 0.03	15.0 ± 0.2	20.2 ± 0.1
**Average**		**7.70 ± 0.06**	**8.20 ± 0.26**	**18.4 ± 3.0**	**25.0 ± 3.7**
PIROL	Control	8.18 ± 0.28	7.32 ± 0.03	37.5 ± 0.6	42.3 ± 0.3
	Supporter	7.92 ± 0.19	7.36 ± 0.04	35.1 ± 0.5	37.3 ± 0.4
	Moncut 460 SC	8.35 ± 0.17	7.50 ± 0.03	31.8 ± 0.1	35.8 ± 0.5
	Supporter +Moncut 460 SC	7.94 ± 0.19	7.57 ± 0.02	24.2 ± 0.3	33.7 ± 0.5
**Average**		**7.40 ± 0.11**	**8.10 ± 0.26**	**32.2 ± 5.3**	**37.3 ± 3.3**
PICUS	Control	7.96 ± 0.27	7.28 ± 0.06	29.9 ± 0.6	38.3 ± 0.7
	Supporter	7.72 ± 0.21	7.29 ± 0.10	28.8 ± 0.3	35.7 ± 0.3
	Moncut 460 SC	8.34 ± 0.22	7.33 ± 0.10	25.4 ± 0.2	33.7 ± 0.6
	Supporter +Moncut 460 SC	7.95 ± 0.19	7.37 ± 0.05	23.3 ± 0.3	29.5 ± 0.6
**Average**		**7.32 ± 0.08**	**7.99 ± 0.29**	**26.9 ± 2.8**	**34.3 ± 3.4**
**Averages for treatments**	Control	8.20 ± 0.27	7.43 ± 0.17	29.8 ± 6.7	36.5 ± 6.0
	Supporter	8.10 ± 0.37	7.45 ± 0.16	28.0 ± 6.6	33.7 ± 4.3
	Moncut 460 SC	8.40 ± 0.23	7.53 ± 0.13	24.5 ± 6.7	30.8 ± 6.0
	Supporter +Moncut 460 SC	8.00 ± 0.23	7.58 ± 0.10	20.8 ± 4.4	27.8 ± 6.0
**Average for the experiment**		**7.50 ± 0.15**	**8.20 ± 0.31**	**25.8 ± 6.9**	**32.2 ± 6.3**
^1^LSD α = 0.05		^2^A = 0.528; B = 0.036; C = 0.047; B/A = 0.051; A/B = 0.529; C/A = 0.067; A/C = 0.530; C/B = 0.082; B/C = 0.080		A = 0.368; B = 0.454; C = 0.259; B/A = 0.642; A/B = 0.631; C/A = 0.366; A/C = 0.473; C/B = 0.449; B/C = 0.597	

^*^AH - Directly after harvest, **AS - After 6 months of storage.

^1^LSD - least significant difference, ^2^Experimental factors: A – Evaluation date, B – Variety, C –Treatment application.

Meanwhile, long-term storage resulted in a decrease in total starch content and an increase in starch acidity (a decrease in pH) in all varieties tested (Tables 1, 2, 7 and 8). This is consistent with reports by Pobereżny et al.^9^, who also obtained an increase in potato starch acidity after long-term storage of tubers of varieties intended for processing, and with the results of Trithavisup and Charoenrein^48^ studying rice starch after 12 months of storage. In addition, starch aging, which results from an increase in acidity and an increase in the proportion of the fraction of grains with the smallest size, significantly deteriorates the texture characteristics of frozen and thawed starch gels - water content after thawing (*r* = 0.641 and *r*=-0.633, respectively) (Table 6) by increasing the amount of water^9^.

### Starch stability after freezing

Wang et al.^50^, Won et al.^51^ and Pobereżny et al.^9^ report that as little water as possible should remain in the starch gel after thawing. The potato varieties studied showed good starch stability after thawing as they had low water content - on average from 25.6% (immediately after tuber harvest) to 32.1% (after 6 months of storage) (Tables 7 and 8). Thus, these values were at a much lower level than in studies by other authors^51–53^. In our earlier study^17^, the water content of starch from three varieties for processing was, on average, at 34.2% (immediately after harvest) and at 35.5% (after storage). In contrast, as reported by Won et al.^51,^ Wang et al.^52^ and Srichuwong et al.^53^, water content after thawing can be up to 76.0%, which is unfavorable for potatoes destined for refined products. Freezing causes physical stress which is often detrimental to starch gels. It can also result in the tearing of starch granules, especially when the residual moisture content is high^54^.

### Gelatinization temperature

The process of starch gelatinization is widely used in the food industry. It is a complex phenomenon involving the destruction of ordered granule structures and the full completion of the process occurs when the starch loses its crystalline structure. Therefore, this process is characterized by such indicators of starch quality as the onset and end gelatinization temperatures^42,50,55,56^. Furthermore, the functionality of starch is closely related to its hydration level^50,56^. In this study, the gelatinization temperature depended on the genetic conditions of the potato (Tables 9 and 10), which is consistent with the results obtained by other authors^57,58^. The onset and end gelatinization temperatures in this study averaged from the years of study, respectively: 63.1 and 67.1 °C. The range of onset and end gelatinization temperatures of potato starch was comparable to the results obtained by Skansberger and Kocherbitov^59^, respectively: 61.7 and 69.9 °C. It should be noted that Kovač et al.^58^, studying 8 potato genotypes, obtained significant differences in the onset gelatinization temperatures of starch for the varieties used in the experiment. Moreover, dos Santos et al.^57^ and Kovač et al.^58^ obtained significant differences between potato genotypes for the end gelatinization temperature of starch. In addition, it was noted that in the studies of dos Santos et al.^57^ and Kovač et al.^58^, the onset and end gelatinization temperatures for starch were at a higher level than in this study (Tables 9 and 10). The difference in the obtained values of gelatinization temperatures may be due to the intended use of the tested potato genotypes, as our varieties are intended for refined products while those of dos Santos et al.^57^ and Kovač et al.^58^ are for consumption and starch production, respectively. According to Neeraj et al.^5^, the gelatinization temperature for potato starch in addition to genotype, is also influenced by the timing of testing (storage time). It was shown that the onset and end gelatinization temperatures were at higher levels than immediately after tuber harvesting and such effect was obtained in each year of the study (Tables 9 and 10). The increase in gelatinization temperatures was respectively: 0.3 and 1.1 °C. This is due to an increase in the fraction of small grains after tuber storage whose gelatinization temperatures increase^60^. This is confirmed by the highly significant negative correlation coefficients calculated in this study between the size of starch grains and the onset and end gelatinization temperatures after storage: *r*=-0.585 and *r*=-0.395, respectively (Table 6). According to Dhital et al.^55^, the end gelatinization temperature of potato starch decreases with increasing starch grain size while the onset gelatinization temperature is not affected by starch grain size. Skansberger and Kocherbitov^59^ take a different view claiming that the onset temperature of starch gelatinization increases with increasing fraction of the largest grains. In contrast, the end gelatinization temperature decreases for these starch grains. According to the authors^61,62^, starch grains with smaller diameters have a higher surface area-to-weight ratio so they absorb water and swell faster than large grains, thus lowering the gelatinization temperature. The influence of temperature during tuber storage on the value of starch gelatinization temperature was also reported by Kaur et al.^45^. The authors obtained an increase in starch gelatinization temperatures after storing tubers for a period of four months at higher temperatures (8, 12, 16 and 20 °C). On the other hand, Neeraj et al.^5^ report that there is a decrease in starch gelatinization temperatures at higher tuber storage temperatures. However, these authors’ studies involved half the storage period compared to this study.

**Table 9:** Gelatinization temperature of starches from tested potato variety depending on treatments and evaluation date in 2021.

Variety	Treatment	Gelatinization temperature [℃]			
		AH*		AS**	
		Onset	End	Onset	End
BEO	Control	63.1 ± 0.1	68.4 ± 0.2	62.9 ± 0.1	68.7 ± 0.1
	Supporter	62.6 ± 0.3	66.8 ± 0.1	62.7 ± 0.1	67.6 ± 0.2
	Moncut 460 SC	61.1 ± 0.1	65.6 ± 0.1	62.5 ± 0.1	67.7 ± 0.1
	Supporter +Moncut 460 SC	61.0 ± 0.2	65.3 ± 0.1	62.4 ± 0.1	66.8 ± 0.2
**Average**		**64.0 ± 0.7**	**68.4 ± 0.8**	**64.1 ± 0.8**	**68.7 ± 0.7**
PIROL	Control	65.0 ± 0.1	69.6 ± 0.1	65.1 ± 0.1	69.6 ± 0.2
	Supporter	64.0 ± 0.2	68.0 ± 0.2	64.6 ± 0.1	69.0 ± 0.2
	Moncut 460 SC	63.8 ± 0.1	68.3 ± 0.2	63.4 ± 0.1	68.3 ± 0.1
	Supporter +Moncut 460 SC	63.1 ± 0.1	67.7 ± 0.1	63.3 ± 0.1	67.8 ± 0.2
**Average**		**62.0 ± 1.0**	**66.5 ± 1.3**	**62.6 ± 0.2**	**67.7 ± 0.7**
PICUS	Control	63.8 ± 0.2	68.3 ± 0.1	63.8 ± 0.2	69.2 ± 0.3
	Supporter	64.1 ± 0.1	65.9 ± 0.3	62.9 ± 0.1	68.2 ± 0.1
	Moncut 460 SC	61.9 ± 0.1	65.4 ± 0.1	62.6 ± 0.1	67.5 ± 0.3
	Supporter +Moncut 460 SC	61.0 ± 0.3	65.1 ± 0.2	62.4 ± 0.3	66.8 ± 0.2
**Average**		**62.7 ± 1.4**	**66.2 ± 1.3**	**62.9 ± 0.6**	**67.9 ± 0.9**
**Averages for treatments**	Control	64.0 ± 1.1	68.8 ± 1.3	63.9 ± 0.5	69.2 ± 0.5
	Supporter	63.6 ± 1.2	66.9 ± 1.4	63.4 ± 0.4	68.3 ± 0.4
	Moncut 460 SC	62.3 ± 0.7	66.4 ± 0.9	62.8 ± 0.9	67.8 ± 0.6
	Supporter +Moncut 460 SC	61.7 ± 0.8	66.0 ± 0.6	62.7 ± 1.0	67.1 ± 0.4
**Average for the experiment**		**62.9 ± 1.3**	**67.0 ± 1.5**	**63.2 ± 0.9**	**68.1 ± 0.9**
^1^LSD α = 0.05		Onset gelatinization temperature: ^2^A = 0.235; B = 0.131; C = 0.112; B/A = 0.185; A/B = 0.274; C/A = 0.158; A/C = 0.265; C/B = 0.194; B/C = 0.213		End gelatinization temperature: A = 0.208; B = 0.116; C = 0.127; B/A = 0.164; A/B = 0.243; C/A = 0.180; A/C = 0.253; C/B = 0.220; B/C = 0.223	

^*^AH - *Directly after harvest, **AS - After 6 months of storage.

^1^LSD - least significant difference, ^2^Experimental factors: A – Evaluation date, B – Variety, C –Treatment application.

**Table 10:** Gelatinization temperature of starches from tested potato variety depending on treatments and evaluation date in 2022.

Variety	Treatment	Gelatinization temperature [℃]			
		AH*		AS**	
		Onset	End	Onset	End
BEO	Control	63.3 ± 0.1	68.0 ± 0.1	62.8 ± 0.1	68.5 ± 0.1
	Supporter	63.0 ± 0.2	67.2 ± 0.2	63.1 ± 0.1	68.2 ± 0.1
	Moncut 460 SC	61.5 ± 0.1	66.0 ± 0.1	62.9 ± 0.1	67.5 ± 0.2
	Supporter +Moncut 460 SC	61.4 ± 0.1	65.7 ± 0.2	62.8 ± 0.1	67.4 ± 0.2
**Average**		**62.3 ± 0.7**	**66.7 ± 0.5**	**62.9 ± 0.8**	**67.9 ± 0.7**
PIROL	Control	65.4 ± 0.2	69.2 ± 0.3	65.5 ± 0.1	69.6 ± 0.1
	Supporter	64.4 ± 0.1	68.4 ± 0.1	65.0 ± 0.1	69.4 ± 0.2
	Moncut 460 SC	64.4 ± 0.1	68.1 ± 0.2	63.8 ± 0.1	68.4 ± 0.1
	Supporter +Moncut 460 SC	63.5 ± 0.4	67.9 ± 0.1	63.7 ± 0.2	68.1 ± 0.1
**Average**		**64.4 ± 0.9**	**68.4 ± 1.0**	**64.5 ± 0.2**	**68.9 ± 0.5**
PICUS	Control	64.4 ± 0.2	67.9 ± 0.3	64.2 ± 0.1	69.0 ± 0.2
	Supporter	63.7 ± 0.1	66.3 ± 0.1	63.3 ± 0.1	68.8 ± 0.1
	Moncut 460 SC	62.3 ± 0.2	65.8 ± 0.2	63.0 ± 0.3	67.3 ± 0.2
	Supporter +Moncut 460 SC	61.6 ± 0.2	65.5 ± 0.2	62.8 ± 0.1	67.4 ± 0.2
**Average**		**63.0 ± 1.2**	**66.4 ± 1.0**	**63.3 ± 0.6**	**68.1 ± 0.8**
**Averages for treatments**	Control	64.4 ± 1.0	68.4 ± 1.3	64.2 ± 0.5	69.0 ± 0.5
	Supporter	63.7 ± 1.3	67.3 ± 1.0	63.8 ± 0.4	68.8 ± 0.4
	Moncut 460 SC	62.7 ± 0.6	66.6 ± 0.9	63.2 ± 0.9	67.7 ± 0.6
	Supporter +Moncut 460 SC	62.2 ± 0.9	66.4 ± 06	63.1 ± 1.2	67.6 ± 0.4
**Average for the experiment**		**63.2 ± 1.3**	**67.2 ± 1.2**	**63.6 ± 0.9**	**68.3 ± 0.8**
^1^LSD α = 0.05		Onset gelatinization temperature: ^2^A = 0.158; B = 0.080; C = 0.114; B/A = 0.113; A/B = 0.179; C/A = 0.161; A/C = 0.205; C/B = 0.197; B/C = 0.188		End gelatinization temperature: A = 0.299; B = 0.116; C = 0.093; B/A = 0.164; A/B = 0.322; C/A = 0.131; A/C = 0.314; C/B = 0.161; B/C = 0.181	

^*^AH - Directly after harvest, **AS - After 6 months of storage.

^1^LSD - least significant difference, ^2^Experimental factors: A – Evaluation date, B – Variety, C –Treatment application.

The application of products for potato seed treatment resulted in a reduction in both onset and end gelatinization temperatures in each year of the study (Tables 9 and 10). The lowest gelatinization temperatures were obtained after the combined application of Supporter and Moncut 460 SC, respectively: 62.0 and 66.2 °C. This tendency was also maintained after long-term storage of the tubers. Pycia et al.^62^, on the other hand, did not obtain an increase in the gelatinization temperature of potato starch following the application of biofertilizer. It should be noted, however, that the biofertilizer in the study by Pycia et al.^62^ was applied to the soil and not to the potatoes. In addition, the literature indicates^62,63^ that lower air temperature during growing season leads to an increase in the gelatinization temperatures of starch extracted from potatoes. Such a correlation was demonstrated in this study as there was a lower average air temperature during potato cultivation in 2022 compared to 2021. In 2022, higher onset and end gelatinization temperatures of potato starch were obtained, which reached 63.2 and 67.2 °C, respectively (Table 10). Singh et al.^64^ observed that differences in temperature values during starch gelatinization are significantly related to their degree of crystallinity. In a later study, Singh et al.^65^, studying starch from potatoes grown in India, showed a negative correlation between the degree of amylopectin polymerization and the onset and end gelatinization temperatures. Our findings showed negative correlation coefficients (*p* < 0.05) between amylose content in starch and its gelatinization temperatures: onset *r*=-0.244 and end *r*=-0.251 (Table 5); which is also confirmed by the negative correlations between amylose content and starch gelatinization temperatures obtained in other authors’ studies^66^. The differences between the results of Singh et al.^65^, ours and authors^66^ may be due to the botanical origin of the starch and the environmental conditions in which the potatoes were grown.

### Amylose content in starch

**Fig. 1:**
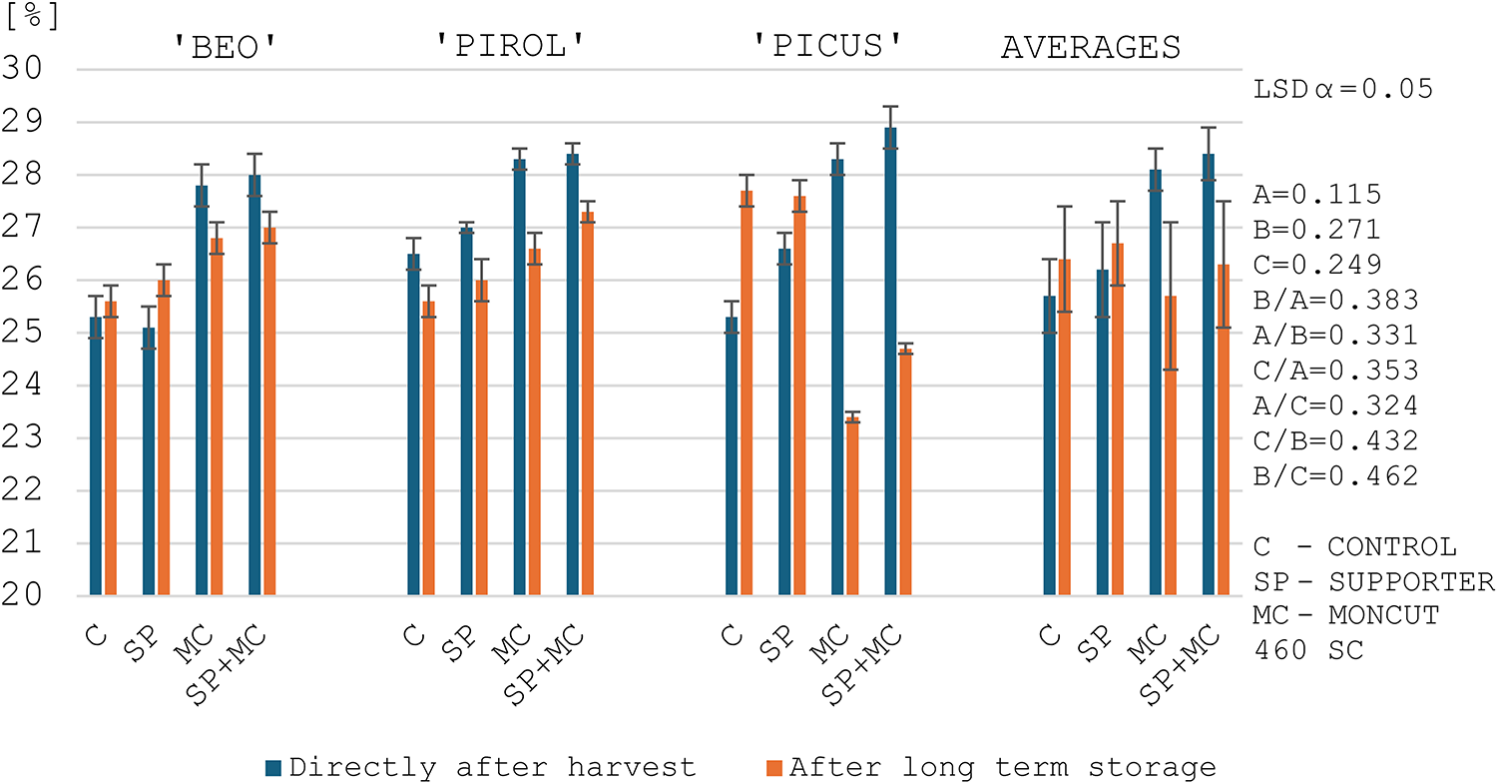
Amylose content in the starch of the tested potato varieties depending on the treatments and the evaluation date in 2021. ^1^LSD - least significant difference, ^2^Experimental factors: A – Evaluation date, B – Variety, C –Treatment application.

**Fig. 2:**
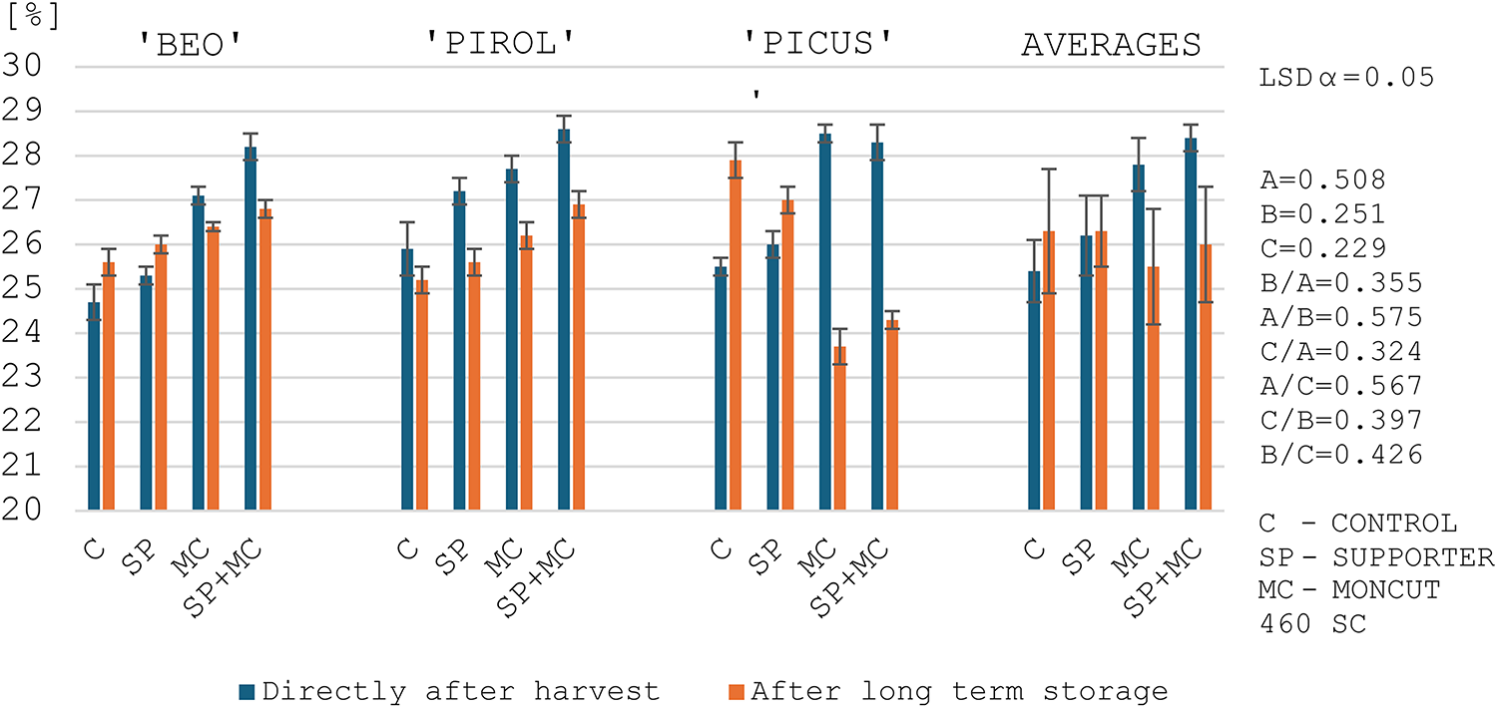
Amylose content in the starch of the tested potato varieties depending on the treatments and the evaluation date in 2022. ^1^LSD - least significant difference, ^2^Experimental factors: A – Evaluation date, B – Variety, C –Treatment application.

Starches vary in amylose content depending on botanical origin and even within the range of the same plant varieties grown in different conditions^10,44,57^. In this study, the post-harvest amylose content of three potato varieties ranged from 24.7 to 28.9% with an average of 27.0% (Figs. 1 and 2). According to Tong et al.^60^, the amylose content in potato starches ranges from 11.4 to 36.6%, and according to Singh et al.^67^ from 6.5 to 32.2%. Jansky and Fajardo^44^, studying 9 potato varieties, obtained amylose contents ranging from 28.9 to 31.0%. On the other hand, Ahmed et al.^68^ studying 29 potato varieties obtained amylose content in potato starch from 18.9 to 29.4% with an average of 25.3%. Šimková et al.^35^, Jansky and Fajardo^44^ and Ahmed et al.^68^ observed a significant impact of environmental conditions and the year of potato cultivation on amylose content in starch. Hu et al.^69^, in their study, noted an increase in amylose content in potato starch caused by an increase in temperature during the growing season. This research confirms such a correlation, as potatoes grown in 2021 contained more amylose than in 2022 with a lower average temperature (Figs. 1 and 2).

The treatments used in the experiment had a significant effect on the increase of amylose content in potato starch (Figs. 1 and 2). The highest content was obtained after combined application of both products. The applied treatments may have influenced a decrease in the activity of enzymes responsible for starch synthesis, which may have resulted in an increase in the amylose content of potato starch^70–72^. The impact of the technology used in cultivation on amylose content is reported by Singh et al.^67^, Ebúrneo et al.^73^ and Zhang et al.^72^. In the study of Ebúrneo et al.^73^, differential nitrogen fertilization did not change the amylose content of starch, while Zhang et al.^72^ showed a significant effect of potassium fertilization, upon application of which the amylose content decreased. However, there is limited information on the influence of modulators on the amylose content of potato tubers.

After long-term storage, the amylose content of starch significantly decreased, by an average of 0.9 pp, regardless of the year and other experimental factors (Figs. 1 and 2). The lowest decrease in amylose content was observed in the Beo variety (0.2 pp), and the highest in the Picus variety (1.4 pp). Jansky and Fajardo^44^ report that the amylose content in starch extracted from stored potato tubers depended on the air temperature during storage. According to the authors, at 4 °C, the amylose content in starch was lower − 29.5% as compared to the content determined at a tuber storage temperature of 6 °C − 30.6%. Furthermore, Labuschagne et al.^74^ and Pobereżny et al.^9^ state that the amylose content in starch after storage also depends on the length of the storage period. According to the authors the longer the storage period, the greater the decrease in amylose content in starch. In addition, increasing temperature during storage enlarges these losses. Labuschagne et al.^74^ also state that the reduction in amylose content during storage is due to its partial breakdown by amylase, and there may also be a transformation of the elongated shape of amylose to one of two helix structures. On the other hand, Singh et al.^75^ showed little change in amylose content after 6 months of storage.

### Phosphorus content in starch

**Fig. 3:**
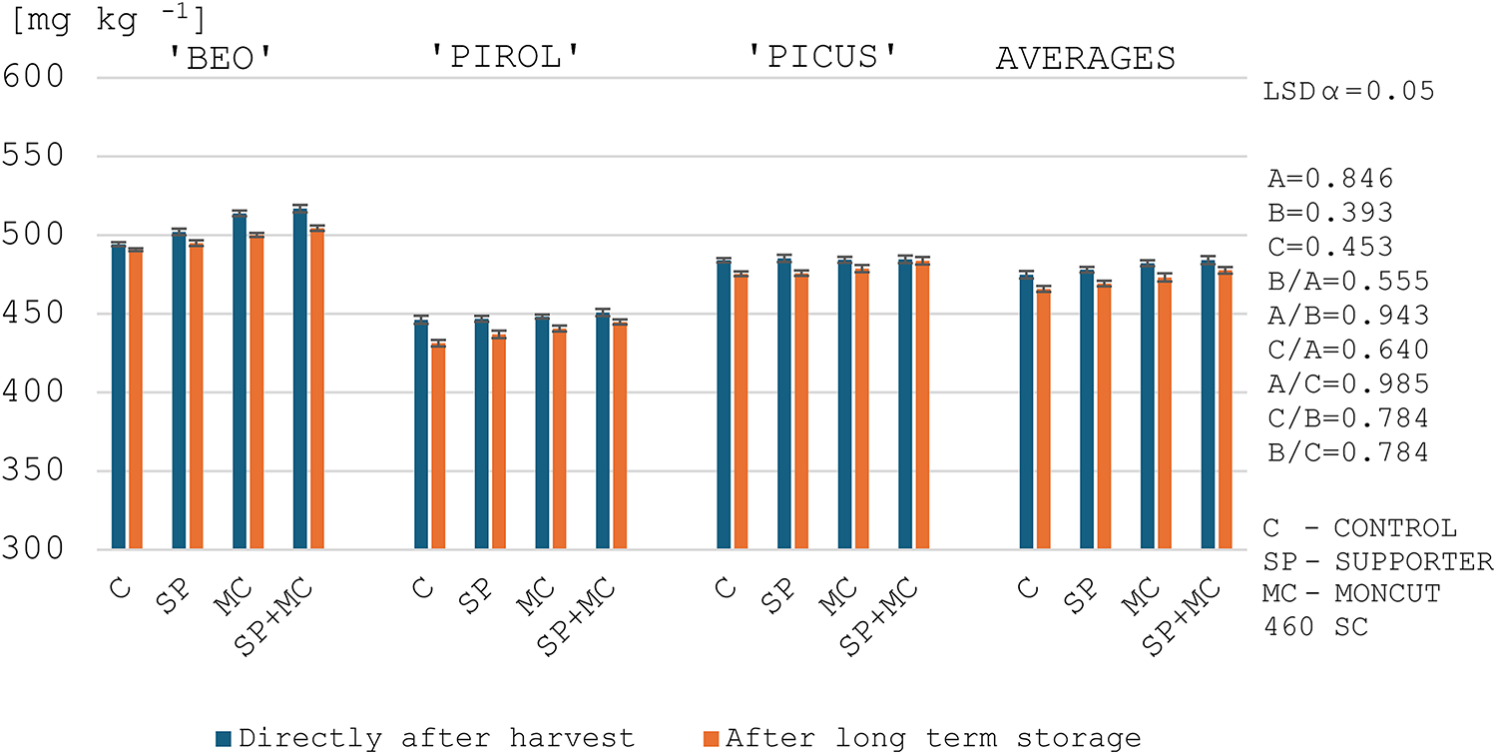
Phosphorus content in the starch of the tested potato varieties depending on the treatments and the evaluation date in 2021. ^1^LSD - least significant difference, ^2^Experimental factors: A – Evaluation date, B – Variety, C –Treatment application.

**Fig. 4:**
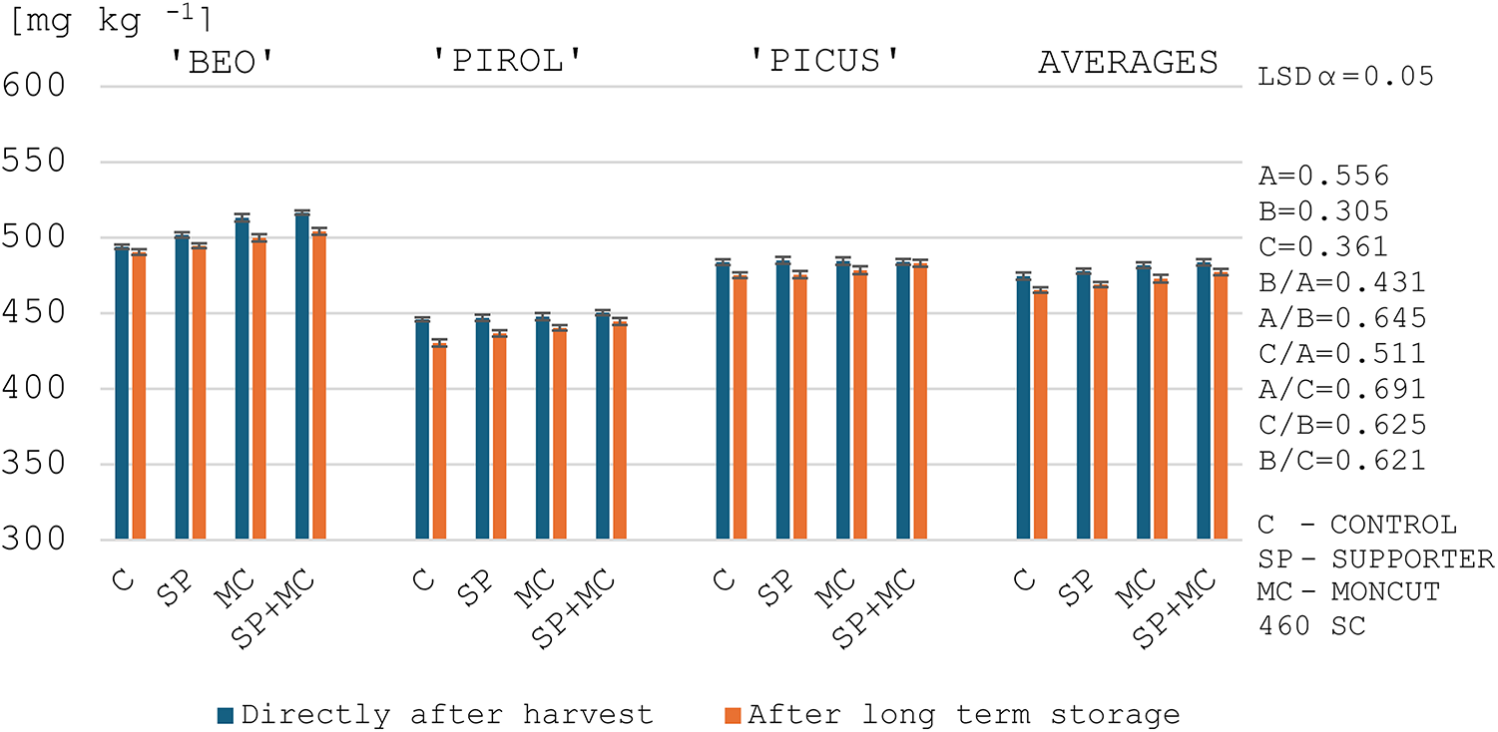
Phosphorus content in the starch of the tested potato varieties depending on the treatments and the evaluation date in 2022. ^1^LSD - least significant difference, ^2^Experimental factors: A – Evaluation date, B – Variety, C –Treatment application.

Since phosphorus measurement in potato starch is an important indicator for its use in the production of certain food products, this research also included this indicator of starch quality. In this study, the phosphorus content in the starch obtained from the tubers after harvest, regardless of the year of the study, ranged from 446.0 to 516.8 mg kg^− 1^, and the varieties studied differed significantly in their phosphorus levels (Figs. 3 and 4). Noda et al.^76^ and Noda et al.^77^ testing potato starches sourced from 69 to 535 potato varieties found phosphorus contents ranging from 621 to 1090 mg kg^− 1^ and from 308 to 1244 mg kg^− 1^, respectively. Ezekiel et al.^46^ determined that phosphorus content of potato starch ranged from 700 to 900 mg kg^− 1^. The results obtained by the aforementioned authors were generally higher than in this study (Figs. 3 and 4). The difference may be due to genetic conditions – their influence on phosphorus content in starch has also been reported by other authors^17,35^.

In addition, the applied treatments caused an increase in the phosphorus content of starch (Figs. 3 and 4). It was noted that the increase in the phosphorus content occurred alongside a decrease in the gelatinization temperature (Tables 9 and 10; Figs. 3 and 4). Dhital et al.^55^ report that high phosphorus levels in starch are attributed to a reduction in its gelatinization temperature. This is confirmed by results of this study, as highly significant negative correlation coefficients were obtained between gelatinization temperatures (onset and end) and phosphorus content, *r*=-0.538 and *r*=-0.487, respectively (Table 5). The reason for this is that negatively charged phosphate groups cause charge repulsion and reduce the tendency for inter-chain bonding, facilitating the hydration of starch granules during heating^55^. In contrast, Noda et al.^77^ obtained a slight significant positive correlation between the temperature of gelatinization and the phosphorus content of starch. The difference in the obtained correlations between our own research and the report by Noda et al.^77^ may be due to the genotypes grown and different environmental conditions. In addition, the authors^77^ do not specify the purpose of the potatoes studied, their earliness group and their starch content.

After long-term storage of tubers, there was a decrease in phosphorus content in starch by an average of 1.7% (Figs. 3 and 4). Ezekiel et al.^46^ and Wszelaczyńska et al.^20^ generally obtained a significant increase in phosphorus content in starch after storage. The authors report that the level of phosphorus in starch depends on storage conditions, mainly air temperature. Starch isolated from tubers stored at higher temperatures contains higher amounts of phosphorus. Meanwhile, in a study by Pobereżny et al.^9^, starch from potatoes stored at 2–6 °C contained an average of 1.5% more phosphorus than starch from tubers tested immediately after harvest.

## Conclusion

Among the tested potatoes for food processing, the Beo variety had the highest starch content and its best quality characteristics. The use of treatments significantly affected the physicochemical properties of potato starch. After the combined application of the products, the tested potato varieties had the highest starch content, which had the best starch stability after freezing, and the lowest onset and end gelatinization temperatures. The changes caused by the application of treatments can be attributed to an increase in the proportion of large starch grains and higher amylose and phosphorus content. After long-term storage of tubers, the quality of starch decreased slightly. This was mostly influenced by maintaining constant conditions during tuber storage along with using products for potato seed treatment. For potatoes intended for food processing and starching, it should be recommended to apply products for potato seed treatment, as this is the cheapest and most effective form of potato protection. Due to the wide range of starch uses, the research conducted should be an area of great interest. However, it should be taken into account that the starch of plants of the same botanical variety and even of the same plant after cultivation under different environmental conditions may not only have different structures but also perform different functions. In view of the above, it is recommended to expand the research on the effect of the applied cultivation and storage technology on the properties of starch of potatoes grown under other environmental conditions and to include research with varieties with other uses. In addition, due to the continuous increase in demand for starch, it is recommended to expand the research to other plant species.

## Materials and methods

### Research material

The subjects of the study were tubers of three varieties of potato (*Solanum tuberosum* L.) intended for processing into chips: ‘Beo’, ‘Pirol’ and ‘Picus’ from Potato Breeding Norika Poland Ltd. Characteristics of the tested varieties: ‘Beo‘(early, average dry matter content − 23.9% starch − 18.5% in fresh tuber weight); ‘Pirol’ (medium early, average dry matter content − 24.1%, starch − 17.8% in fresh tuber weight); ‘Picus’ (medium late, average dry matter content − 24.0%, starch − 18.3% in fresh tuber weight).

In general, a three-factor experiment was established (2021/2022, 2022/2023):

- Evaluation date (immediately after harvest; after 6 months of storage);
- Potato variety (‘Beo’, ‘Pirol’, ‘Picus’);
- Application of treatments immediately before planting: control (no application of treatments); Supporter (main ingredient - synthetic amino acids) − 0.3 dm^3^ ha^− 1^; Moncut 460 SC (main ingredient - *flutolanil*) − 0.2 dm^3^ t^− 1^; combined application of treatments (Supporter 0.3 dm^3^ ha^− 1^+ Moncut 460 SC 0.2 dm^3^ t^− 1^).

### Field experiment

A two-factor field experiment (potato varieties, application of treatments) was established using the randomized sub-block method in three repetitions. The field experiment was located in the Agricultural Experiment Station in Tomaszkowo (53º42’N, 20º26’E, Poland) belonging to the University of Warmia and Mazury in Olsztyn.

The field experiment was conducted on a brown soil proper made of strong loamy sands. This soil is classified as class IV b of the 5 - good rye complex.

Before the field experiment began, the soil was characterized by medium levels of available phosphorus (P) and magnesium (Mg) and high levels of potassium (K). The soil was characterized by an acid reaction (Table 11). The soil’s P content was determined by colorimetric method, K by atomic emission spectrometry (AES), and Mg by atomic absorption spectrophotometry (AAS)^78^. Organic carbon content was determined by the Tiurin method in mineral soil samples^79^. Soil reaction was determined using a digital pH meter.

**Table 11:** Soil parameters before the field experiment (2021, 2022).

Growing season	pH	C_org_ (g kg^− 1^)	Available macronutrients (mg kg^− 1^)		
			*P*	K	Mg
2021	5.3	12.7	66.71	157.1	58.0
2022	5.0	15.8	51.01	290.5	79.0

Potatoes were planted at a row spacing of 0.625 × 0.3 m. The size of the one plot for planting and harvesting was 5.6 m^2^ (1.87 m x 3 m). The planting depth was 7 cm. A ridge forming aggregate was used to obtain a trapezoidal shape. The forecrop of potato in the study years was oats.

No organic fertilization was applied to the crop. Mineral fertilization was applied: 120 N kg ha^− 1^ (urea 46% N) - before planting 80 N kg ha^− 1^ and until the end of plant emergence (immediately before forming ridges) 40 kg ha^− 1^; K_2_O 160 kg ha^− 1^ (potassium sulfate 50%) and P_2_O_5_ 80 kg ha^− 1^ (granular triple superphosphate 40%) - considering the nutrient content of the soil. Mineral fertilizers P and K were applied to the soil immediately before planting potatoes. Potato planting was carried out in the first decade of May in the study years (2021, 2022).

All agrotechnical procedures and the use of plant protection products were carried out in accordance with the principles of proper agrotechnics.

In order to reduce weed infestation, double ridging (forming ridges) was used.

The tuber yield was harvested at full maturity (depending on the earliness group). Samples of tubers were taken from each experimental site for analytical tests immediately after harvest (10 kg) and for long-term storage (10 kg). Medium-sized tubers were stored in chambers (Thermolux Refrigeration Air Conditioning, Raszyn, Poland) with controlled atmosphere for 6 months (October-March). A constant temperature of + 8 °C and relative humidity of 95% recommended for potatoes intended for food processing were maintained throughout the storage period.

### Meteorological conditions

Tables 3 and 4 show meteorological conditions (air temperature and precipitation) during the potato growing seasons (2021, 2022).

Weather conditions during the potato growing season were more favorable in 2021 as the average air temperature (15.8 °C) and total precipitation (494.6 mm) were higher compared to 2022.

The temperatures that occurred during the 2021 and 2022 growing seasons were optimal for the potato in every month except May in which the temperature should exceed 14.0 °C. In 2021, the highest temperature was in July (average month 20.8 °C) with high precipitation (178.4 mm). In contrast, in 2022 the highest temperature occurred in August (average month 20 °C) with low precipitation (29.6 mm).

In potato cultivation, it is most favorable when high precipitation occurs in July and August, which was the case during the 2021 field experiment (178.4 and 136.4 mm, respectively).

### Evaluation of potato starch quality

#### Procedure for determination of starch content in dry matter of potato tubers

The percentage of starch content in the dry matter of potato tubers was calculated on the basis of dry matter and starch content in the fresh weight of tubers.

Calculation:$$\:PR\:=\:\frac{SC\:\:\:}{DMC}\times\:100\:\left(\%\right)$$

PR – percentage of starch in dry matter (%).

SC – starch content in fresh weight of tubers (g kg^− 1^).

DMC – dry matter content of tubers (g kg^− 1^).

Determination of dry matter content in potato tubers was made according to AACC International Methods^80^ while starch content was determined according to the polarimetric Evers method^81^.

#### Starch preparation

Samples consisting of 5 kg of medium-sized potato tubers were used for starch preparation. The tubers were washed thoroughly with distilled water and then cut into small pieces. The cut sample was homogenized in a mixer with distilled water. Then, using metal sieves with mesh sizes of 300 and 150 μm, the prepared suspension was filtered twice. The filtrate (starch suspension) was left to stand for 2 h, and then washed three times with distilled water Then, the starch grains were recovered from the extraction by decanting. The obtained starch was dried at 40 °C in a dryer (WAMED, model SUP-100, Warsaw, Poland) to a constant moisture content of less than 20%. Purified starch samples were stored in a dry place at + 20 °C until analysis.

#### Procedure for determination of starch grain size

Starch samples (0.2–1 g) were weighed, transferred quantitatively to a 50-ml Erlenmeyer flask, and then 25 ml of distilled water was added. Using the method of sedimentation of starch in water, the starch was separated into small and large fractions. The large fraction of potato starch was removed after five minutes and the small fraction was removed after a 90-minute sedimentation period. Starch granularity (grain size distribution) was determined using a Fritsh Analysette 22 laser analyzer (Idar-Oberstein, Germany)^82^.

#### Procedure for determination of total starch content

The determination of the total starch content was carried out polarimetrically (Krüss, type P 1000, Hamburg, Germany). For this purpose, 2.5 g of potato flour was weighed and then quantitatively transferred to a 100 ml conical flask. Then 50 ml of 1.124% HCl (Chempur, Piekary Śląskie, Poland) was added and the whole thing was shaken thoroughly for 30 min. The flask was covered and then placed in a boiling water bath for 15 min, stirring occasionally. After cooling to 20 ℃, the solution was quantitatively transferred to a 100 ml volumetric flask, 7 ml of 10% Wolframatophosphoric acid (POCH, Gliwice, Poland) was added, and the whole was mixed thoroughly. The flask was made up to 100 ml with distilled water and mixed again. The solution was then filtered through a No. 593 ½ paper filter (Schleicher & Schuell, Taufkirchen, Germany) discarding the first drops of the filtrate. The filtrate was placed in a polarimetric tube and a reading was taken^83^.

#### Procedure for determination of potato starch pH

25 g of potato starch was weighed into a 100 ml beaker. Then 25 ml of distilled water was added and the whole mixture was mixed thoroughly. The resulting starchy milk was left to stand for 15 min and stirred occasionally. While stirring, the pH was determined by reading the value from a pH meter scale (HI2002-02, Hanna Instruments, Woonsocket RI, USA)^84^.

#### Procedure for determining the stability of starch after thawing

A filtration method was used to determine the volume of water after thawing the starch solution. The starch suspension (5%) was heated at 95 °C for 30 min. The starch solution was then cooled at room temperature for 30 min. Then, 30 g of the solution was weighed out and frozen in centrifuge tubes at -20 °C for 22 h (Freezer-WHIRLPOOL, AFG 6402 E-B, Comerio, Italy). The post-thaw stability of the starch was evaluated after thawing the samples in a water bath at 50 °C for 90 min and centrifugation (Hettina Zentrifugen, Rotina 420 R, Germany) at 1000 rpm for 15 min. This parameter was calculated as the amount (%) of H_2_O separated from the original starchy milk mass^85^.

#### Procedure for determination of starch gelatinization temperature

To determine the onset and end temperatures of starch gelatinization, 4 g of starch was weighed into a glass beaker. 100 ml of distilled water at room temperature was added. The content of the beaker was stirred to obtain starch milk. The beaker was placed in a boiling water bath. The following was then measured: the onset and end temperatures of starch gelatinization using a laboratory thermometer with a range of -50 ℃ to + 300 ℃ (TFA Dostmann 30.1048, Wertheim, Germany.)^86^.

#### Procedure for the determination of amylose content in starch

Amylose content was determined by staining starch samples with I_2_-KI and measuring absorbance at 600 nm. 25 mg of the starch sample was mixed with 0.5 ml of 45% HClO_4_ (Merck KGaA, Germany). After 4 min, 8 ml of distilled water was added. Staining with I_2_-KI was performed by mixing 4 ml of starch solution with 5 ml of Lugol’s solution (Merck KGaA, Germany) diluted with distilled H_2_O (1:3). Absorbance was measured in a spectrophotometer (SHIMADZU UV-1800, UV Spectrophotometer System, Japan) immediately after mixing^87^.

#### Procedure for determination of phosphorus content in starch

Starch samples for phosphorus estimation were digested in a mixture of H_2_SO_4_ and H_2_O_2_ in an automatic Digest Automat K-438, K371 (BUCHI Labortechnik AG, Switzerland). Phosphorus content in starch was measured spectrophotometrically (SHIMADZU UV-1800, UV Spectrophotometer System, Japan) as inorganic phosphorus (according to the ammonium-molybdate method) for further calculation of phosphorus content in starch samples according to the modified Noda method. In the volumetric flask were placed: 1 ml of mineralized starch sample, 2 ml of 20% Na_2_SO_4_, 2 ml of 2.78% solution of (NH₄)₂MoO4, 2–3 drops of 8.6% SnCl_2_ and 50 ml of distilled water. After 30 min, the absorbance was measured at 660 nm. Chemical reagents were provided by POCH, Gliwice, Poland^63^.

### Statistics

The results generated in the study were subjected to statistical calculations, and significant differences were evaluated with Tukey’s multiple confidence intervals for a significance level of *P* < 0.01 and *P* < 0.05. Analysis of variance of the data was conducted using Statistica 13.1 software (StatSoft, Tulsa, OK, USA). Mean scores and standard deviation are presented in tables and figures. To get a synthetic picture of the overlapping relationships between the studied characteristics, a simple correlation analysis (Pearson) was performed.

## Data Availability

The datasets used and/or analysed during the current study are available from the corresponding author(s) on reasonable request.
