# Author Correction: Potato starch quality in relation to the treatments and long-term storage of tubers

**DOI:** 10.1038/s41598-025-94324-3

**Published:** 2025-03-20

**Authors:** Katarzyna Brążkiewicz, Jarosław Pobereżny, Elżbieta Wszelaczyńska, Bożena Bogucka

**Affiliations:** 1 Institute of Agri-Foodstuff Commodity, Bydgoszcz University of Science and Technology, 7 Kaliskiego St, 85-796 Bydgoszcz, Poland; 2 Department of Agrotechnology and Agribusiness, University of Warmia and Mazury in Olsztyn, 8 Oczapowskiego St, 10-719 Olsztyn, Poland

Correction to: *Scientific Reports,* 10.1038/s41598-025-85657-0, published on 3 February 2025.

The Funding section in the original version of this Article was omitted. The Funding section now reads:

The results presented in this paper were obtained as part of a comprehensive study financed by the University of Warmia and Mazury in Olsztyn, Faculty of Agriculture and Forestry, Department of Agrotechnology and Agribusiness (grant No.30.610.013–110).

The original Article has been corrected.

